# LSnet: detecting and genotyping deletions using deep learning network

**DOI:** 10.3389/fgene.2023.1189775

**Published:** 2023-06-14

**Authors:** Junwei Luo, Runtian Gao, Wenjing Chang, Junfeng Wang

**Affiliations:** School of Software, Henan Polytechnic University, Jiaozuo, China

**Keywords:** structural variation, deletion, convolutional neural network, attention mechanism, gated recurrent units network

## Abstract

The role and biological impact of structural variation (SV) are increasingly evident. Deletion accounts for 40% of SV and is an important type of SV. Therefore, it is of great significance to detect and genotype deletions. At present, high accurate long reads can be obtained as HiFi reads. And, through a combination of error-prone long reads and high accurate short reads, we can also get accurate long reads. These accurate long reads are helpful for detecting and genotyping SVs. However, due to the complexity of genome and alignment information, detecting and genotyping SVs remain a challenging task. Here, we propose LSnet, an approach for detecting and genotyping deletions with a deep learning network. Because of the ability of deep learning to learn complex features in labeled datasets, it is beneficial for detecting SV. First, LSnet divides the reference genome into continuous sub-regions. Based on the alignment between the sequencing data (the combination of error-prone long reads and short reads or HiFi reads) and the reference genome, LSnet extracts nine features for each sub-region, and these features are considered as signal of deletion. Second, LSnet uses a convolutional neural network and an attention mechanism to learn critical features in every sub-region. Next, in accordance with the relationship among the continuous sub-regions, LSnet uses a gated recurrent units (GRU) network to further extract more important deletion signatures. And a heuristic algorithm is present to determine the location and length of deletions. Experimental results show that LSnet outperforms other methods in terms of the F1 score. The source code is available from GitHub at https://github.com/eioyuou/LSnet.

## 1 Introduction

Structural variations (SVs), small insertions and deletions (indels), and single nucleotide polymorphisms (SNPs) make up the majority of genetic variations in humans. The sizes of SVs range from fifty to ten thousand bps or even more. They mainly include five types: deletions, insertions, inversions, duplications and translocations (Sudmant et al., 2015). According to previous research, there are thousands of SVs in each human genome (Collins et al., 2020), with deletions corresponding to the highest proportion of approximately 40%, and inversions corresponding to the lowest proportion of only 1% (Lei et al., 2022). Their roles and biological impact are becoming increasingly obvious, and increasing evidence is showing the importance of SVs in all biological categories and conditions, such as cancer (Aganezov et al., 2020), Alzheimer’s disease and autism (Beyter et al., 2021). Undoubtedly, accurate detection of structural variants is important, but it remains an unsolved problem.

The development of sequencing technology is particularly important for efficient SV detection in the genome. Sequencing technology has evolved from Sanger sequencing (Sanger et al., 1977) to next-generation short read sequencing technology (NGS) (Eid et al., 2009), and then to long read sequencing technology (Carneiro et al., 2012), which directly sequences individual DNA molecules. The sequencing lengths of short reads are usually in the range of 150–500 bp. Short read sequencing is popular and cost effective SV detection strategy. Short read sequencing has a high accuracy rate, and the error rate can be as low as 1%. The errors are mainly small insertions/deletions (indels) and base substitutions. However, despite its advantages, it has certain difficulties when dealing with complex regions, such as repeated regions and regions with high or low GC content. Long read (third generation) sequencing technology is represented by Oxford Nanopore Sequencing of Oxford Nanopore Technologies (ONT) (Clarke et al., 2009) and single molecule real time (SMRT) technology (Carneiro et al., 2012) of Pacific Biosciences (PacBio). The reads obtained with third generation sequencing technology are longer, but the accuracy rate is lower. The 5%–20% error rate of long read sequencing technology is significantly higher than that of NGS (less than 1%). However, these error-prone long reads are more suitable for detecting longer SVs and can handle complex regions better. Circular consensus sequencing (CCS) improves the accuracy of SMRT sequencing (PacBio) and generates highly accurate (99.8%) long high fidelity (HiFi) reads with an average length of 13.5 kilobases (kb) (Wenger et al., 2019). It can detect SVs well.

Sequencing based methods are commonly used for detecting SVs, with good detection accuracy and sensitivity. Based on the type of sequencing data used, SV detection methods can be divided into short read alignment based approaches and long read alignment based approaches.

Short read alignment based approaches typically call SVs based on read depth, read pairs, or split reads. The read depth refers to the calculated number of reads covering a base in the reference genome. The read pairs method detects SVs by considering whether a read pair has an abnormal information, such as insert size, alignment direction, alignment location. When a read covers an SV, the read is usually split into two or more small fragments for alignment, and the SV can be detected based on the split positions. For example, DELLY (Rausch et al., 2012), LUMPY (Layer et al., 2014), and Manta (Chen et al., 2016) detect SVs by integrating two or three of the above characteristics for improving accuracy.

At present, SV detection methods using long reads mainly rely on intra-read and inter-read alignment signatures. Intra-read alignment signatures enable the direct prediction of an SV based on a large alignment gap between the reference and a read. Inter-read alignment signatures involve inconsistencies in position, size, and orientation among multiple long reads, similar to short read signatures. Common methods include, cuteSV (Jiang et al., 2020), sniffles (Sedlazeck et al., 2018), SVIM (Heller and Vingron, 2019) and pbSV. These methods use both inter alignment signatures and intra alignment signatures, but the ways in which they call SVs are different. Sniffles analyzes SVs based on multiple features, such as split read alignment, statistical analysis of mismatched regions, and read depth. PbSVs are detected by calculating the lengths of variant clusters and the consistency of variant sites. SVIM uses graph based clustering methods and a new SV signature distance metric to cluster detected signatures. CuteSV uses a variety of extraction methods to comprehensively collect the signatures of various SVs. PBSVs are detected using heuristic algorithms and specially designed clustering and refinement methods.

In recent years, deep learning has greatly improved the state of the art in visual detection, speech recognition, and many other fields. There are already some approaches using deep learning to call SVs. DeepVariant (Poplin et al., 2018) uses convolutional neural networks (CNNs) for SNP and small indel calls. It constructs candidate variant site images for classification and has been shown to outperform all state-of-the-art variant callers. DeepSV (Cai et al., 2019) also uses a deep learning approach for the problem of calling SVs. It uses a new visual sequence read method to call long deletions. Both DeepVariant and DeepSV use short read data to call SVs. BreakNet (Luo et al., 2021) is a new approach for detecting deletions using long read. It builds a matrix by extracting the information of the “D” operations in a CIGAR string and uses a CNN and recurrent neural network to call deletions. However, BreakNet cannot determine the genotype of a deletion.

In this study, we introduce LSnet, an approach for detecting and genotyping deletions with deep learning networks. LSnet uses the combination of error-prone long reads and short reads or HiFi reads, and it can effectively use deep learning to automatically detect deletions of different sizes and determine their genotypes. LSnet overcomes the problem of the high sequencing error rate of long reads based on the high accuracy of short reads. Experimental results show that LSnet achieves better deletion detection results than current popular SV callers.

## 2 Materials and methods

**FIGURE 1:**
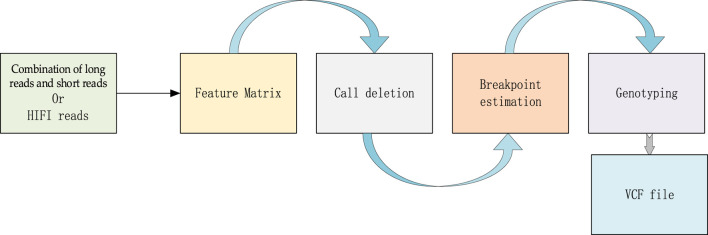
The workflow of LSnet.

LSnet is a deletion detecting and genotyping method, which can not only adopt the combination of error-prone long reads and short reads as input, but also high accurate long reads (HiFi reads). It is divided into two modules: a deletion call module and a genotype call module. In the deletion call module, there are three main steps i) In the first step, LSnet divides the reference genome into continuous sub-regions. Based on the alignment file, LSnet extracts the alignment features in each sub-region to generate feature matrixes. ii) In the second step, LSnet uses a CNN and an attention module for feature extraction. Next, LSnet uses a bidirectional gated recurrent unit (GRU) module to analyze the feature relationship among sub-regions and determines whether a sub-region represents a variation through a fully connected layer. iii) The third step is to call the exact breakpoint based on the CIGAR strings and a heuristic algorithm. In the genotype call module, LSnet uses the same feature matrix as input, and uses a CNN model to call the genotype. The overall workflow of LSnet is shown in Figure 1.

### 2.1 Generating feature matrix

LSnet uses a sorted BAM file as the input to extract read alignment features. LSnet divides the reference genome into continuous sub-regions with L (200 bp in default) in length. Then, for one position of a sub-region, LSnet extracts nine features and records them as a 9-tuple SIG (read_lc_, read_ld_, read_llb_, read_lrb_, read_lsp_, read_lm_, read_sc_, read_slb_, read_srb_). read_lc_ denotes the long read coverage in this position. read_ld_ denotes the number of long reads that indicate this position as a deletion. read_llb_ and read_lrb_ denote the numbers of long reads indicating this position as a split position (llb means that the left part of the read can be aligned with the reference genome while the other part is soft-clipped; lrb means that the right part of a read can be aligned with the reference genome and the other part is soft-clipped). read_lsp_ denotes the number of split alignments at this position for long reads. read_lm_ denotes the average coverage of each 10 million bp length region of the chromosome. read_sc_ denotes the short read coverage in the position. read_slb_ and read_srb_ denote the numbers of short read indicating this position as a split position. Then, LSnet uses Formula (1) to normalize the elements in the SIG.

$$\begin{array}{c} {SIG}_{long\_read\left(i\right)}=\frac{{Sig}_{long\_read\left(i\right)}-{SIG}_{long\_read\;mean}}{{SIG}_{long\_read\;std}}\\ {SIG}_{short\_read\left(j\right)}=\frac{{SIG}_{short\_read\left(j\right)}-{SIG}_{short\_read\;mean}}{{SIG}_{short\_read\;std}} \end{array}$$ (1)

SIG_long_read_ includes six elements, read_lc_, read_ld_, read_llb_, read_lrb_, read_lsp_, read_lm_, and SIG_longread(i)_ is the *i*th element in SIG_long_read_. SIG_long_read_mean_ represents the average of these six elements. SIG_long_read std_ denotes the standard deviation of SIG_long_read_. SIG_short_read_ includes three elements, read_sc_, read_slb_, read_srb_, and SIG_short_read(j)_ is the *j*th element in SIG_short_read_. SIG_short_read mean_ represents the average of these three elements. SIG_short_read std_ denotes the standard deviation of SIG_short_read_. Each position in a sub-region includes the above nine features. LSnet can generate a corresponding feature matrix with nine rows and L columns for each sub-region. If a sub-region is less than 200 bp, the matrix is padded with 0.

### 2.2 Building neural network

**FIGURE 2:**
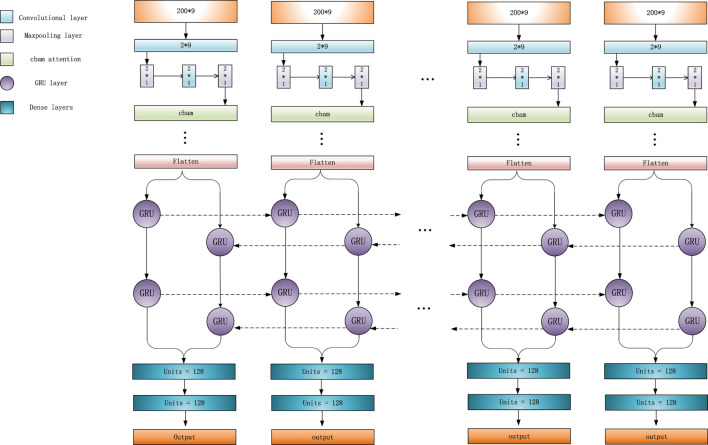
LSnet module for detecting deletions.

When a region referred to a variant, its adjacent normal regions usually display different characteristics. So, the relationships between adjacent regions can effectively help detect variations. For example, in a region corresponding to a deletion, the number of “D” in CIGAR string should be much higher than that in its adjacent normal regions, and the read coverage of this deletion region should be lower than that of an adjacent normal region. At the same time, if the length of a deletion exceeds L, a single feature matrix cannot completely represent this deletion. So, LSnet performs feature extraction from the feature matrix and then captures the features of the relationships among adjacent sub-regions to identify deletions. According to previous research, CNN models (Kattenborn et al., 2021) are very effective for feature extraction. Therefore, LSnet performs feature extraction on the original features by means of a CNN. Moreover, spatial and channel attention mechanisms are added to enable the network to notice more critical features and extract more important information. In terms of capturing the relation-ship features among adjacent sub-regions, Gated Recurrent Units (GRU) (Cho et al., 2014)networks yield similar experimental results with long short term memory (LSTM) (Yu et al., 2019) networks, but GRU networks are easier to compute. Therefore, LSnet captures the features among continuous sub-regions from two directions by means of a bidirectional GRU network to allow the model to better detect deletion region. Finally, the sub-regions are predicted through two fully connected layers. The network structure is shown in Figure 2.

**FIGURE 3:**
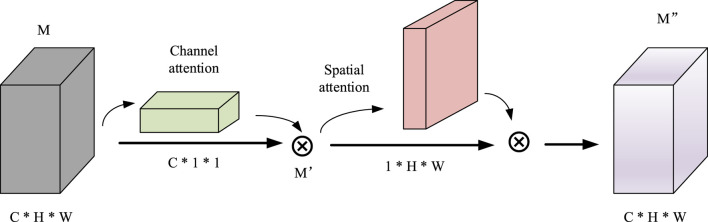
The architecture of CBAM.

In detail, the CNN consists of two dimensional convolution, maximum pooling and attention mechanism modules. First, after four convolutions, a convolutional block attention module (CBAM) (Woo et al., 2018) is added. Since the convolution operation extracts informative features by mixing cross channel and spatial information together, important features are emphasized along the two main dimensions, i.e., the channel and spatial axes, by CBAM. CBAM sequentially applies channel and spatial attention modules (shown in Figure 3) to increase the expressiveness of the features by means of an attention mechanism, focusing on important features and suppressing unnecessary features. C, H, and W in Figure 3 represent the number of channels, length, and width of this feature, respectively.

CBAM is a simple and effective attention module that is widely applied to improve the representational ability of CNNs. The specific calculation formula is shown in Formula (2).

$$\begin{array}{c} M^{\prime }=Channel\left(M\right)⊗M\\ M^{\prime \prime }=Spatial\left(M^{\prime }\right)⊗M^{\prime } \end{array}$$ (2)

The features M after convolution and maximum pooling areas is used as input, and the final result $M^{\prime \prime }$ is obtained through a one dimensional channel attention module Channel and a two dimensional spatial attention module Spatial. The ⊗ indicates elementwise multiplication.

LSnet uses a bidirectional GRU network to integrate the information generated at different time steps. This bidirectional GRU neural network uses a total of two 64 pairs of Gated Recurrent Units, which can process the hidden representations generated by the CNN module in a forward and reverse manner.

Finally, three fully connected layers are used for prediction. The first two fully connected layers each have a dropout layer (0.4) behind them, and the last fully connected layer uses the sigmoid function as the activation function. If the final output result is greater than 0.5, the associated sub-region is considered as a deletion; otherwise, it is not a deletion.

### 2.3 Breakpoint estimation

After the sub-regions are predicted by the previous modules, LSnet determines the exact starting and ending positions of all deletions based on the sub-regions that are identified as deletions. Each sub-region predicted as deletion is recorded as a triple (chr, ref_start_, ref_end_). chr indicates which chromosome it belongs to, and ref_start_ and ref_end_ indicate the starting and ending positions on the reference genome where the deletion is located. Then, from the CIGAR string of each read, LSnet finds the position whose “D” > 30 bp within the sub-region. If the following Formula (3) is satisfied, it will be recorded as a sub-signature. cigar_start_ indicates the starting position of the deletion in the CIGAR string of the read, and cigar_length_ indicates the length of the deletion in the CIGAR string. If the distance between two sub-signature is less than 20 bp, they will be merged into one long deletion region.

$$\begin{array}{c} \left|{cigar}_{start}-{ref}_{start}\right|\;<\;200\\ {cigar}_{length}\;>\;30 \end{array}$$ (3)

Due to error-prone long reads and the design of the alignment tools, a large deletion may be split into multiple smaller parts in the reference. Then, LSnet finds the split alignment based on the soft-cliped and hard-cliped positions in the CIGAR string. The starting and ending positions of the read and the starting and ending positions of the reference are recorded.

**FIGURE 4:**
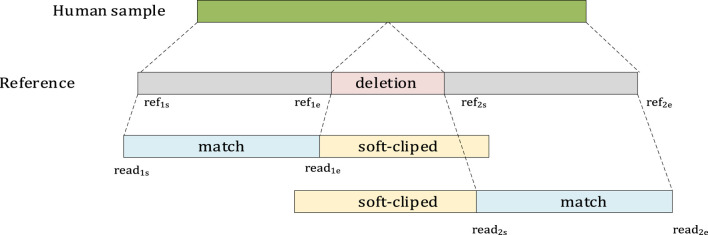
An example of soft-clipped alignment.

As shown in Figure 4, if two segments seg_1_ and seg_2_ belong to the same read, and they are adjacent on the reference and are aligned with the chromosomes in the same direction, then Distance is calculated. If Distance >40 bp, it is considered a deletion. ref_2s_ and read_2s_ indicate the starting positions of the reference and read, respectively, in seg_2_. reF1_e_ and read_1e_ indicate the ending positions of the reference and read, respectively, in seg_1_.

$$Distance=\left({ref}_{2s}-{ref}_{1e}\right)-\left({read}_{2s}-{read}_{1e}\right)$$ (4)

The regions found above are all recorded in the form of a triple (chr, rref_start_, rref_end_), where rref_start_, and rref_end_ denote the starting and ending positions, respectively, on the reference. All regions found are sorted. If the two regions are on the same chromosome and the two regions satisfy the following Formula (5), it is considered that the two reads represent the same deletion variation region and are stored in the same bin. After all data have been processed, the final starting and ending positions of each deletion are determined in accordance with the starting positions of all data in each bin and the median deletion length.

$$\begin{array}{c} {rref}_{start2}-{rref}_{start1}\;<=1500\\ {rref}_{end2}-{rref}_{end1}\;<=1500 \end{array}$$ (5)

### 2.4 Genotyping

**FIGURE 5:**
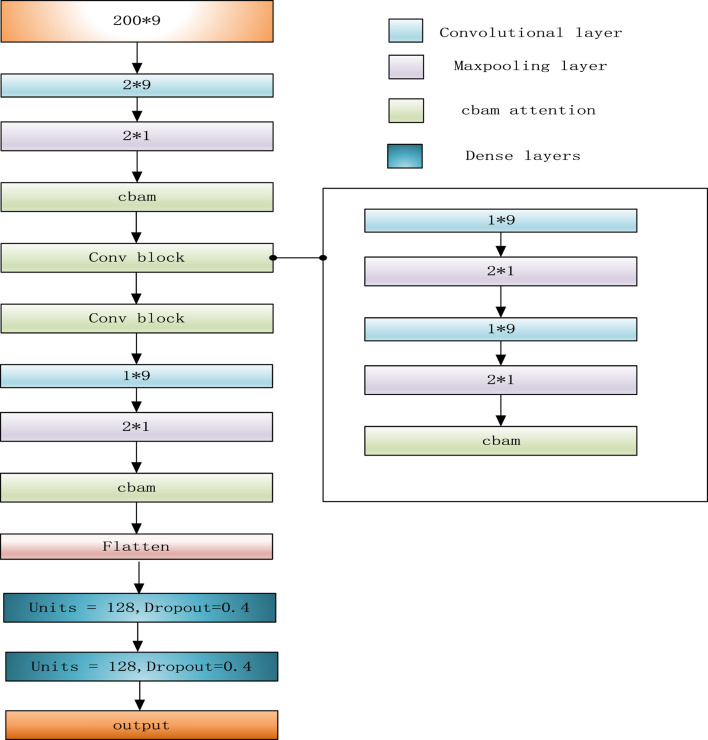
LSnet module for detecting genotype.

In the previous step, we obtained specific location information about each deletion. To detect the specific genotype of each deletion, LSnet adopts the network structure shown in Figure 5. The genotype prediction model uses only a CNN and an attention mechanism. The network structure is similar to that of the deletion call model, and the same data are used as for deletion detection. If the result is less than 0.5, the deletions considered a heterozygous variant and is marked as (0, 1). If the result is greater than 0.5, it is considered a homozygote and is marked as (1, 1). If there is a large variation, it will be covered by multiple variant feature matrices. For such a case, we count all the prediction results for the component subregions and select the prediction result with the highest frequency as the genotype.

### 2.5 Model training

Data collected with different sequencing technologies were used, including PacBio CCS, PacBio CLR long read HG002 dataset, and 2 × 250 bp short read data collected by the Illumina platform HG002. For CLR data with relatively low accuracy, LSnet uses both HG002 CLR long read data and HG002 Illumina platform short read data to obtain an accurate feature matrix. Due to the high accuracy of CCS data, short read data were not used. The high confidence call set from the Genome in a Bottle Consortium (GIAB) was used to label the data, produce training, testing and validation dataset. This call set is widely used by other callers to evaluate the efficiency of SV. Deletion re-gions were set to 1, and other regions were set to 0. After the data were labeled, they were divided into training, testing, and validation sets for training and evaluation. The data of chromosomes 1–10 were used as training data, the data of chromosome 11 were used as validation data, and the data of chromosomes 12–22 were used as testing data. We implemented all callers on a computer with a 12 core, 24 thread CPU (Intel(R) Xeon(R) Silver 4214 CPU @ 2.20 GHz). We used a single RTX 3090 video card for model training.

### 2.6 Model predicting

When performing variant detection, to adequately detect all variant sites, we ran the detection process two times. The first detection was for normal subregions, for example, [0–200], [200–400], and [400–600]. The results obtained were the sub-regions of potential variant sites. The second detection, performed with a 100 bp sliding window, generated new data subregions, for example, [100–300], [300–500], and [500–700]. The data were then fed back into the neural network for deletion prediction. If a variation was found in a sub-region in the second prediction but not in the first, the variant sub-region would be located in the overlap between the results of the first and second predictions. LSnet marks both parts of the normal region as deletion. For example, if [300–500] is a deletion, LSnet marks [200–400], [400,600] two regions as deletions. This approach can effectively improve the prediction results.

## 3 Results

The high-confidence SVs collected by GIAB were employed as the ground truth, and LSnet was benchmarked against five long reads SV callers and two short reads SV callers. The long reads SV callers include sniffles (2.0.6), SVIM (1.4.2), pbSV (2.6.2.), cuteSV (1.0.13), and BreakNet (2.0). And tow short reads callers include Manta (1.6.0), Delly (1.1.5). Truvari was used to assess the results and record the precision, recall and F1 score. Five well studied human samples were chosen: HG002 CLR (mean read length: 7,938 bp), HG002 CCS (mean read length: 13,478 bp), HG002 Illumina (mean read length: 250 bp), NA19240 (mean read length: 6,503 bp), and NA19240 Illumina (mean read length: 120 bp). The details of the datasets are shown in Supplementary Table S1 in the Supplementary Materials. In the HG002 dataset, chromosomes 11–22 were used as the testing dataset for LSnet, and in the NA19240 dataset, chromosomes 1–22 were used as the testing dataset. At the same time, the corresponding short read data from GIAB were used, downsampled to the same coverage. The support read parameters of SIVM, cuteSV, and LSnet were set to 10, 5, 3, 3, and 2 for the HG002 CLR 69X, 35X, 20X, 10X and 5X datasets, and the support read parameters of SVIM, cuteSV, and LSnet were set to 3, 2, and 1 for the HG002 CCS 28X, 10X, and 5X datasets. We set the support read parameters of the SV callers to 10 and 3 for the NA19240 CLR 40X and 10X datasets. Deletions smaller than 50 bp were removed.

### 3.1 Deletion detection with CLR HG002 data

First, we benchmarked LSnet, sniffles, SVIM, cuteSV, Pbsv, BreakNet, Manta, Delly on the HG002 CLR 69X chromosome 12–22 data. The benchmark results for sample HG002 are shown in Table 1. LSnet achieved the highest precision, recall and F1 score for the 69X dataset. Next, we randomly downsampled the HG002 chromosome 12–22 data to 35X, 20X, 10X, and 5X and tested the performance of the SV callers on these datasets with different coverages. As shown in Table 1, LSnet achieved the best F1 score for all coverages. On the 35X downsampled data, LSnet also maintained precision, recall, and F1 scores higher than those of the other tools. On the 20X and 10X downsampled data, sniffles and cuteSV, respectively, achieved the highest precision. On the 5X downsampled data, LSnet had the lowest precision, but its recall was 14% higher than that of the second best method. This proves that LSnet achieves better performance than other SV callers under different coverages.

**TABLE 1:** The performance comparison of SV callers on CLR dataset about HG002.

	Depth		LSnet	Sniffles	SVIM	cuteSV	pbSV	breaknet	Delly	Manta
Hg002clr	69X	precision	0.9737	0.9598	0.9659	0.9733	0.9601	0.9695	0.4808	0.7163
		recall	0.9517	0.9554	0.9411	0.9373	0.9637	0.9131	0.577	0.5702
		F1	0.9626	0.9576	0.9533	0.955	0.9619	0.9405	0.5245	0.635
	35X	precision	0.9712	0.9608	0.9597	0.9692	0.9477	0.9371	0.6001	0.7588
		recall	0.9411	0.9434	0.935	0.9282	0.9449	0.9335	0.4796	0.7396
		F1	0.9559	0.952	0.9472	0.9483	0.9463	0.9353	0.5332	0.5567
	20X	precision	0.963	0.9591	0.9622	0.9627	0.9396	0.9658	0.692	0.7824
		recall	0.9048	0.9041	0.9033	0.8958	0.9169	0.8527	0.3716	0.315
		F1	0.933	0.9308	0.9318	0.928	0.9281	0.9057	0.4835	0.4491
	10X	precision	0.96	0.9588	0.9791	0.9825	0.919	0.9782	0.738	0.7727
		recall	0.7983	0.7908	0.673	0.6775	0.8225	0.6767	0.1745	0.1156
		F1	0.8718	0.8667	0.7977	0.802	0.8681	0.8	0.2822	0.2011
	5X	precision	0.7773	0.9699	0.9673	0.9704	0.9412	0.5248	0.7105	0.8167
		recall	0.6775	0.5113	0.5355	0.5438	0.5438	0.7621	0.0612	0.037
		F1	0.724	0.6696	0.6894	0.697	0.6893	0.6217	0.1127	0.0708

### 3.2 The performance for deletions with different deletion sizes

We also benchmarked the results of the long read SV callers for different deletion sizes on the 69X dataset, as shown in Table 2. We set sizes of [50–200], [200–500], [500–1,000], and [1,000,]. LSnet achieved relatively high F1 scores. LSnet had the highest precision on the 50 and 200 bp data and the third highest recall. This shows that LSnet can accurately find deletions sites in small variant regions. On regions larger than 1,000 bp, LSnet had the highest precision and recall, thus proving that LSnet is more sensitive to SVs in large regions.

**TABLE 2:** The performance of SV callers in different deletion size about HG002 69X data.

Size		Sniffles	cuteSV	pbSV	SVIM	LSnet	BreakNet
50–200	precision	0.943	0.9575	0.9509	0.9515	0.9599	0.9614
	recall	0.9313	0.8984	0.9533	0.9162	0.9203	0.9231
	F1	0.9371	0.927	0.952	0.9335	0.9397	0.9418
200–500	precision	0.9692	0.9663	0.9543	0.9714	0.9765	0.9612
	recall	0.9793	0.9663	0.9741	0.9663	0.9689	0.9637
	F1	0.9742	0.9663	0.9641	0.9688	0.9727	0.9625
500–1,000	precision	0.9841	1	0.9841	0.9219	0.9683	0.9531
	recall	0.9841	0.9365	0.9841	0.9365	0.9683	0.9683
	F1	0.9841	0.9672	0.9841	0.9391	0.9683	0.9606
1,000-	precision	0.9728	0.9797	0.9718	0.9792	0.98	1
	recall	0.9597	0.9732	0.9262	0.9463	0.9866	0.651
	F1	0.9662	0.9764	0.9485	0.9625	0.9833	0.7886

### 3.3 The results of LSnet with support reads

We assessed the support read parameter in LSnet on HG002 CLR datasets with various coverages (69X, 35X, and 20X). The support read parameter indicates the minimum number of supporting reads for an SV to be reported. As shown in Table 3, setting different numbers of support reads leads to different results. When the set number of support reads was larger, the precision was improved, and the sensitivity was reduced. When a smaller number of support reads was set, the precision decreased, and the sensitivity increased.

**TABLE 3:** The performance of different support read on HG002 data.

Coverage		Support>=1	Support>=3	Support>=5	Support>=10
69X	precision	0.9662	0.9597	0.9648	0.9737
	recall	0.9524	0.9524	0.9517	0.9517
	F1	0.9442	0.956	0.9582	0.9626
35X	precision	0.9441	0.9644	0.9712	0.9712
	recall	0.9441	0.9418	0.9411	0.9411
	F1	0.9441	0.953	0.9559	0.9559
20X	precision	0.9216	0.963	0.9715	0.9824
	recall	0.9147	0.9048	0.8746	0.7175
	F1	0.9181	0.933	0.9205	0.8293

### 3.4 Deletion detection with NA19240 data

The SV callers were more fully benchmarked by using the PacBio CLR dataset (mean read length, 6,503 bp; coverage, 40X) from another well studied human sample (NA19240). Due to the bamfile of NA19240 lacks a certain parameter, pbsv does not support running on NA19240, we skipped the test of pbsv on NA19240. This dataset contains a total number of 17,950 deletions. The precisions, recalls, and F1 scores of the benchmarked SV callers are shown in Table 4. LSnet obtained good F1 scores. On the 40X data, LSnet obtained an F1 score less than 1% lower than that of the best method, sniffles. Then, we randomly downsampled the data to 10X. On the 10X NA19240 data, LSnet achieved the second highest F1 score.

**TABLE 4:** The performance of SV callers on NA19240 data.

Data	chr		LSnet	Sniffles	cuteSV	SVIM	Delly	Manta
NA19240	1–22	precision	0.9259	0.883	0.9108	8,713	0.5406	0.6941
		recall	0.2209	0.23	0.2238	0.0391	0.1018	0.1152
		F1	0.3566	0.365	0.3594	0.0748	0.1714	0.1975
	1–22	precision	0.905	0.9043	0.9167	0.8451	0.6853	0.8921
		recall	0.2144	0.1637	0.158	0.0549	0.0741	0.2351
		F1	0.3467	0.2772	0.2696	0.1031	0.1337	0.3721

### 3.5 Deletion detection with CCS HG002 data

PacBio CCS data were collected from the same samples of chromosomes 12–22 to further benchmark the SV callers. Due to the high accuracy of CCS data, LSnet did not need to use short read data for assistance. The precisions, recalls, and F1 scores of the benchmarked SV callers are shown in Table 5. LSnet achieved the highest F1 scores on the 30x dataset. Then, we downsampled this dataset to coverages of 10X and 5X. On the 10X downsampled data, the F1 score of LSnet was lower only than that of BreakNet. On the 5X downsampled data, LSnet also achieved the highest F1 scores. In genotype detection, LSnet showed the best performance on all datasets with different coverages. Among them, on the 5X dataset, the F1 score of LSnet was 5% higher than that of the second best method cuteSV.

**TABLE 5:** The performance of SV callers on HG002 CCS data.

Data	Coverage		LSnet	Sniffles	cuteSV	SVIM	pbSV	BreakNet
CCS	28X	precision	0.966	0.9525	0.9486	0.9343	0.9437	0.9518
		recall	0.9456	0.9539	0.9479	0.9554	0.9615	0.9554
		F1	0.9557	0.9532	0.9482	0.9447	0.9525	0.9536
	10X	precision	0.956	0.9552	0.959	0.9455	0.9417	0.9591
		recall	0.9192	0.9026	0.9011	0.9041	0.9509	0.9388
		F1	0.9372	0.9282	0.9291	0.9243	0.9462	0.9489
	5X	precision	0.9637	0.9654	0.9326	0.9223	0.9379	0.962
		recall	0.861	0.6964	0.8776	0.8784	0.8784	0.8414
		F1	0.9095	0.8091	0.9043	0.8998	0.9072	0.8977

### 3.6 The performance comparison of SV caller on genotype

**FIGURE 6:**
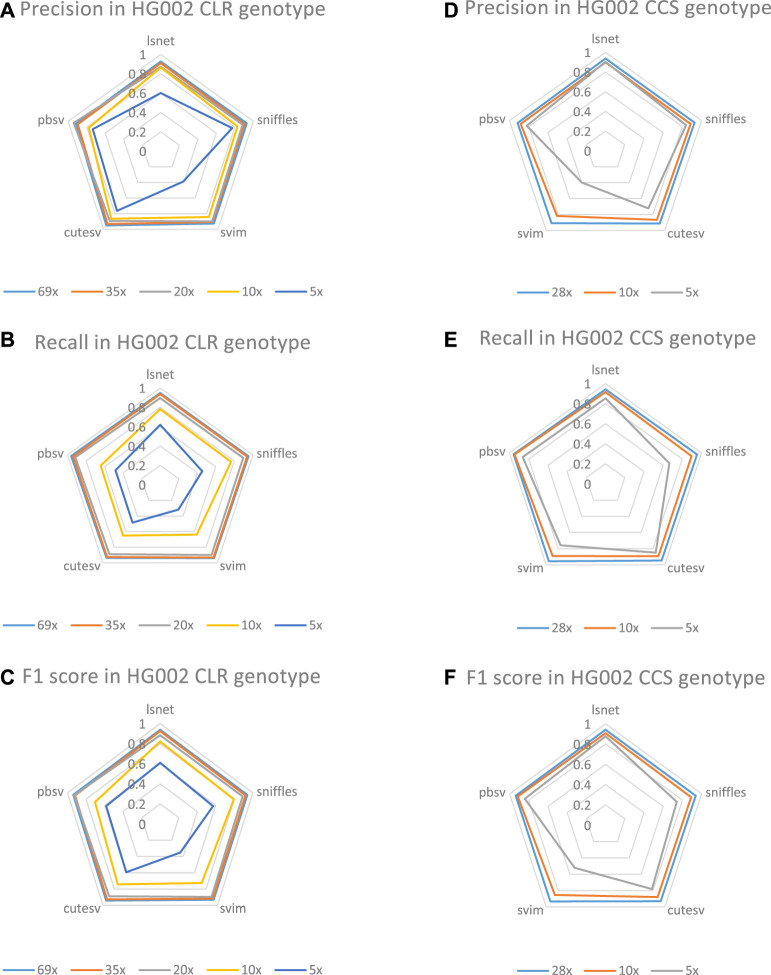
**(A)**The genotype precision on HG002 CLR datasets. **(B)**The genotype recall on HG002 CLR datasets. **(C)**The genotype F1 score on HG002 CLR datasets. **(D)**The genotype precision on HG002 CCS datasets. **(E)**The genotype recall on HG002 CCS datasets. **(F)**The genotype F1 score on HG002 CCS datasets.

We also evaluated the genotype detection ability of LSnet on the CLR and CCS HG002 datasets with different coverages. Regarding the genotyping performance shown in Figure 6, LSnet can achieve good results. Since the short read SV caller menta cannot detect genotypes, delly is relatively poor in deletion detection, so genotype detection is not used. We evaluated genotypes using SV caller for long reads.

In CLR HG002 datasets, as shown in Figure 6, the highest F1 scores were obtained at 35X, 10X and 5X coverage. At the same time, LSnet has the highest precision on 35X, 10X, and 5X. In CCS datasets, we also evaluated the genotype performance of 28X, 10X, and 5X. As shown, LSnet achieves the best F1 score at both 28X and 5X. In 10X dataset, LSnet F1 score is only 0.8% lower than pbSV. And in the 5X data, LSnet improved by 3.6% over the second place pbSV. These results suggest that LSnet also performs well in genotyping.

## 4 Discussion

Due to the high sequencing error rate of long reads and the complexity of SVs, it is still important to take full advantage of the characteristics of both error-prone long reads and accurate short reads. In this study, we developed LSnet to detect deletions using deep learning methods based on the combination of error-prone long reads and short reads or HiFi long reads. LSnet collects different features from long reads and short reads, effectively extracts features through a CNN and an attention mechanism, and then uses a GRU network to mine the relationships among sub-regions to call SVs. We validate LSnet on several well researched datasets and found that it can achieve better performance than other SV callers on data with different coverages.

However, LSnet still has some limitations, which will be important to address in future work to further improve the method. First, LSnet can detect only deletions; however, there are also other types of SVs, such as insertions and translocations. In addition, the run time is a problem to be solved. LSnet needs to extract the features of the data, which takes a long time.

## Data Availability

The original contributions presented in the study are included in the article/Supplementary Material, further inquiries can be directed to the corresponding author.
