# Supplementary material for: LSnet: detecting and genotyping deletions using deep learning network
Source: Front Genet. 2023 Jun 14;14:1189775. doi: 10.3389/fgene.2023.1189775 (PMC10301831; doi:10.3389/fgene.2023.1189775)
Supplement: Supplementary file 1 [file Table1.DOCX]

Supplementary Material

LSnet: detecting and genotyping deletions using deep learning network

Junwei Luo^1^, Runtian Gao^1^, Wenjing Chang^1^, Junfeng Wang^1,*^

*** Correspondence:** Junfeng Wang: luojunwei@hpu.edu.cn

# Dataset

The details information of datasets is described in Table1.

**Table S1. The detail of datasets**

|  | **HG002 CLR** | **HG002 CCS** | **NA19240 CLR** | **HG002 Illumina** | **NA19240 Illumina** |
| --- | --- | --- | --- | --- | --- |
| Read Count | 2915733 | 6596012 | 20452822 | 883914482 | 1857969704 |
| Average | 7938 | 13478 | 6503 | 250 | 120 |
| Coverage | 69X | 28X | 41X | 67X | 74X |
| Aliger | NGMLR | PBMM2 | BWA | BWA | BWA |

# Data Availability

Both the CCS Illumina data and high-confidence call sets for HG002 are available at GIAB FTP site ftp://ftp.ncbi.nlm.nih.gov/giab/ftp/data/AshkenazimTrio/HG002_NA24385_son/.

The Illumina data for NA19240 is available at ftp://ftp.1000genomes.ebi.ac.uk/vol1/ftp/data_collections/hgsv_sv_discovery/data/YRI/NA19240/.

The CLR data for NA19240 is available at http://ftp.1000genomes.ebi.ac.uk/vol1/ftp/data_collections/hgsv_sv_discovery/working/20160905_smithm_pacbio_aligns/NA19240_bwamem_GRCh38DH_YRI_20160905_pacbio.bam

The NA19240 High-confidence call sets are available at NCBI dbVAR: ftp://ftp.ncbi.nlm.nih.gov/pub/ dbVar/data/Homo_sapiens/by_study/vcf/nstd152.GRCh 38.variant_call.vcf.gz.

# Commend lines

Sniffles

sniffles --reference reference.fa --input input.bam --vcf output.vcf

Svim

svim alignment outputpath input.bam reference.fa

CuteSV

cuteSV input.bam reference.fa output.vcf tmppath -t 16 -s (10/5/2/1) --genotype

PBSV

pbsv discover --tandem-repeats .trf.bed inputs.bam output.svsig.gz

pbsv call reference.fa ourput.svsig.gz output.vcf (--ccs) -t INS,DEL

Truvari

Truvari -passonly -p 0 -P 0 –sizefilt 50 –includebeb input.bed -b base.vcf -c compare.vcf -f reference.fa -o outputpath

Samtools

samtools view -s 1234.5 -@ 10 input.bam -o output.bam

# The performance on different data

**Table S2. The performance comparison of SV caller on CLR genotype.**

|  | depth | chr | type |  | lsnet | sniffles | svim | cutesv | pbsv |
| --- | --- | --- | --- | --- | --- | --- | --- | --- | --- |
| HG002 | 69x | 12-22 | genotype | presicion | 0.9266 | 0.9287 | 0.9279 | 0.9514 | 0.9172 |
|  |  |  |  | recall | 0.9493 | 0.954 | 0.9388 | 0.936 | 0.9621 |
|  |  |  |  | F1 | 0.9378 | 0.9412 | 0.9333 | 0.9436 | 0.9391 |
|  | 35x |  |  | presicion | 0.9104 | 0.9038 | 0.907 | 0.9306 | 0.897 |
|  |  |  |  | recall | 0.9374 | 0.94 | 0.9315 | 0.9255 | 0.9419 |
|  |  |  |  | F1 | 0.9239 | 0.9216 | 0.9191 | 0.928 | 0.9189 |
|  | 20x |  |  | presicion | 0.873 | 0.8846 | 0.8978 | 0.892 | 0.9396 |
|  |  |  |  | recall | 0.896 | 0.8968 | 0.8971 | 0.8884 | 0.9169 |
|  |  |  |  | F1 | 0.8844 | 0.8907 | 0.8975 | 0.8902 | 0.9281 |
|  | 10x |  |  | presicion | 0.8656 | 0.8278 | 0.8418 | 0.8653 | 0.7775 |
|  |  |  |  | recall | 0.7811 | 0.7655 | 0.6389 | 0.6491 | 0.6425 |
|  |  |  |  | F1 | 0.8212 | 0.7954 | 0.7264 | 0.7418 | 0.7036 |
|  | 5x |  |  | presicion | 0.6014 | 0.7708 | 0.3902 | 0.7642 | 0.7346 |
|  |  |  |  | recall | 0.6191 | 0.454 | 0.3174 | 0.4842 | 0.482 |
|  |  |  |  | F1 | 0.6101 | 0.5714 | 0.35 | 0.5928 | 0.5821 |

**Table S3. The performance comparison of SV caller on CCS genotype.**

| data | coverage | SV | chr |  | lsnet | sniffles | cutesv | svim | pbsv |
| --- | --- | --- | --- | --- | --- | --- | --- | --- | --- |
| CCS | 30x | genotype | 12-22 | precision | 0.9367 | 0.9276 | 0.9131 | 0.9092 | 0.9158 |
|  |  |  |  | recall | 0.944 | 0.9527 | 0.946 | 0.9543 | 0.9564 |
|  |  |  |  | F1 | 0.9404 | 0.94 | 0.9292 | 0.9312 | 0.9357 |
|  | 10x |  | 12-22 | precision | 0.8987 | 0.8857 | 0.8666 | 0.8175 | 0.8848 |
|  |  |  |  | recall | 0.9145 | 0.8957 | 0.8916 | 0.8907 | 0.9479 |
|  |  |  |  | F1 | 0.9065 | 0.8907 | 0.8789 | 0.8526 | 0.9153 |
|  | 5x |  | 12-22 | precision | 0.9045 | 0.8387 | 0.7231 | 0.3981 | 0.821 |
|  |  |  |  | recall | 0.8533 | 0.6658 | 0.8476 | 0.7572 | 0.8634 |
|  |  |  |  | F1 | 0.8781 | 0.7424 | 0.7804 | 0.5218 | 0.8417 |

|  | **Table S4. Performance comparison of SV callers on CLR dataset about HG002.** | | | | | | | | | | |
| --- | --- | --- | --- | --- | --- | --- | --- | --- | --- | --- | --- |
|  | depth |  | LSnet  (no short read) | LSnet | sniffles | SVIM | cuteSV | pbSV | breaknet | Delly | Manta |
| Hg002clr | 69x | precision | 0.9780 | 0.9737 | 0.9598 | 0.9659 | 0.9733 | 0.9601 | 0.9695 | 0.4808 | 0.7163 |
|  |  | recall | 0.5718 | 0.9517 | 0.9554 | 0.9411 | 0.9373 | 0.9637 | 0.9131 | 0.577 | 0.5702 |
|  |  | F1 | 0.7216 | 0.9626 | 0.9576 | 0.9533 | 0.955 | 0.9619 | 0.9405 | 0.5245 | 0.635 |
|  | 35x | precision | 0.9664 | 0.9712 | 0.9608 | 0.9597 | 0.9692 | 0.9477 | 0.9371 | 0.6001 | 0.7588 |
|  |  | recall | 0.8255 | 0.9411 | 0.9434 | 0.935 | 0.9282 | 0.9449 | 0.9335 | 0.4796 | 0.7396 |
|  |  | F1 | 0.8904 | 0.9559 | 0.952 | 0.9472 | 0.9483 | 0.9463 | 0.9353 | 0.5332 | 0.5567 |
|  | 20x | precision | 0.9676 | 0.963 | 0.9591 | 0.9622 | 0.9627 | 0.9396 | 0.9658 | 0.692 | 0.7824 |
|  |  | recall | 0.8580 | 0.9048 | 0.9041 | 0.9033 | 0.8958 | 0.9169 | 0.8527 | 0.3716 | 0.315 |
|  |  | F1 | 0.9095 | 0.933 | 0.9308 | 0.9318 | 0.928 | 0.9281 | 0.9057 | 0.4835 | 0.4491 |
|  | 10x | precision | 0.9640 | 0.96 | 0.9588 | 0.9791 | 0.9825 | 0.919 | 0.9782 | 0.738 | 0.7727 |
|  |  | recall | 0.7289 | 0.7983 | 0.7908 | 0.673 | 0.6775 | 0.8225 | 0.6767 | 0.1745 | 0.1156 |
|  |  | F1 | 0.8301 | 0.8718 | 0.8667 | 0.7977 | 0.802 | 0.8681 | 0.8 | 0.2822 | 0.2011 |
|  | 5x | precision | 0.8401 | 0.7773 | 0.9699 | 0.9673 | 0.9704 | 0.9412 | 0.5248 | 0.7105 | 0.8167 |
|  |  | recall | 0.5279 | 0.6775 | 0.5113 | 0.5355 | 0.5438 | 0.5438 | 0.7621 | 0.0612 | 0.037 |
|  |  | F1 | 0.6484 | 0.724 | 0.6696 | 0.6894 | 0.697 | 0.6893 | 0.6217 | 0.1127 | 0.0708 |
